# Yemen’s triple emergency: Food crisis amid a civil war and COVID-19 pandemic

**DOI:** 10.1016/j.puhip.2021.100082

**Published:** 2021-01-21

**Authors:** Hashim Talib Hashim, Adriana Viola Miranda, Maryam Salma Babar, Mohammad Yasir Essar, Hasham Hussain, Shoaib Ahmad, Saema Tazyeen, Haya Mohammed Abujledan, Nusaibah Tawfik ALsanabani, Hiba Khan, Mustafa Ahmed Ramadhan, Yahya Dheyaa Tuama, Mashkur Abdulhamid Isa, Attaullah Ahmadi, Don Eliseo Lucero-Prisno, Sheikh Mohammed Shariful Islam, Ashraf Fhed Mohammed Basalilah

**Affiliations:** University of Baghdad, College of Medicine, Iraq; Faculty of Medicine, University of Indonesia, Jakarta, Indonesia; Dubai Medical College, Dubai, United Arab Emirates; Kabul University of Medical Sciences, Kabul, Afghanistan; Allied Hospital, Faisalabad, Pakistan; Punjab Medical College, Faisalabad, Pakistan; Dubai Medical College, Dubai, United Arab Emirates; Department of Genetic Engineering, Hashemite University, Jordan; Department of Molecular Biology and Genetics, Abdullah Gul University, Turkey; Dubai Medical College, Dubai, United Arab Emirates; University of Baghdad, College of Medicine, Iraq; University of Baghdad, College of Medicine, Iraq; School of Health and Related Research, University of Sheffield, United Kingdom; Medical Research Center, Kateb University, Kabul, Afghanistan; Global Health Focus Asia, Kabul, Afghanistan; Department of Global Health and Development, London School of Hygiene and Tropical Medicine, London, United Kingdom; Faculty of Management and Development Studies, University of the Philippines (Open University), Los Banos, Laguna, Philippines; Institute of Physical Activity and Nutrition, Deakin University, Melbourne, Australia; Hadhramaut Hospital, Hadhramaut, Yemen; Ibn Sina General Hospital, Hadhramaut, Yemen

**Keywords:** Food crisis, COVID-19, Yemen, Emergency, Civil war

## Abstract

Yemen has been termed as the world’s worst humanitarian crisis by the United Nations. About 20.1 million (more than 50% of population) Yemenis are facing hunger and 10 million are severely food insecure according to reports by the World Food Programme. With the spread of COVID-19, the situation in Yemen has worsened and humanitarian aid from other countries has become the basis of life for hundreds of thousands of Yemenis after the threat of famine. Yemen is practically one of the poorest countries in the world. It has structural vulnerabilities that have developed over a protracted period of conflict and poor governance and more than 50% live in starving, they suffer for getting one meal a day. To prevent a total collapse of Yemen’s food crises, the government and the international community should act now more decisively.

## 1 Background

The COVID-19 pandemic started from Wuhan, China, and has affected all countries and territories globally [1]. The first case of COVID-19 in Yemen was confirmed on 10th April 2020. Since then, as of 13th December 2020, 2087 confirmed cases and 607 deaths had been reported [2]. However, the real number of cases is likely to be much higher since the instability caused by Yemen’s ongoing civil war and fragile health system has led to limited testing capacity [3]. Epidemiological projections predict that the COVID-19 could infect nearly 16 million (55%) of the Yemeni population [4]. Pandemic-related restrictions have led to devastating socioeconomic consequences in the already war-torn country, including worsening food insecurity. This article sheds light on the food crisis in Yemen amid the civil war and COVD-19 pandemic.

Yemen has been termed as the world’s worst humanitarian crisis by the United Nations (UN). The UN reported that more than 3.6 million Yemenis had been displaced since 2015 due to conflict, famine, multiple diseases, and lack of essential services. According to the World Food Programme (WFP), about 20.1 million (approximately 66%) Yemenis are facing hunger, while 10 million are severely food insecure [5]. Around 2 million children and 1 million women need treatment for malnutrition [6]. The spread of COVID-19 has led to dire effects on Yemeni food security since it causes additional difficulties to acquire food and other goods in the already war-torn country. Before the pandemic, 80–90% of Yemen’s food needs to be imported. The WFP found that due to COVID-19-related restrictions, food imports have decreased by 43% in March 2020, compared to the same month in 2019 [7]. Besides, nearly 25% of vendors reported difficulties in obtaining essential commodities, such as petrol and diesel, with the top 3 COVID-19-related constraints being less availability of imported items, not enough demand and the addition of checkpoints in the supply chain system [8]. These issues have led to increased food prices.

Another key problem is the reduced purchasing power of Yemeni citizens. Even before the pandemic, about 80% of Yemeni were reliant on humanitarian assistance [5]. A survey conducted by the Norwegian Refugee Council found that one in four vulnerable families have lost all their income since the pandemic first hit [9]. In addition, due to a near depletion of foreign exchange reserves, Yemeni local currency is hugely inflated, losing an average of 19% of its value against the US Dollar in the first half of 2020, surpassing the 2018 crisis levels [10]. It is reported that about 20% of Yemenis could not afford sufficient water for their families [9]. Combined with other challenges faced by Yemen, including limited economic opportunities due to the civil war, fragile civil and medical infrastructure, the ongoing economic blockade, less efficient supply chain systems, and inadequate production of electricity, the problems have led to increased Yemen’s vulnerability to famine, food crisis, and infectious diseases amid the pandemic [10].

The international community has initially anticipated this problem to worsen during the pandemic [9]. However, according to UNOCHA, this year’s funding for humanitarian aid is at a historic low, with UN’s 2020 humanitarian appeals being only 25% funded as of September 2020 [7,10]. In June 2020, donor support to UN organizations collapsed, partly due to obstacles and interferences during needs assessments and aid delivery imposed by the Houthi and other Yemeni authorities. Both parties have attempted to redirect the aid exclusive for their supporters [4]. The UN bodies have since then urged for a nationwide ceasefire and continued international assistance since the funding crunch has forced them to halve food assistance for over 9 million Yemenis [4,11].

## 2 Conclusion

With the dwindling humanitarian aid, the ongoing war and the COVID-19 pandemic, Yemen is facing a triple emergency [12]. To prevent a complete collapse of Yemen’s humanitarian crisis, the Yemeni government and international community should act more decisively and implement rules and regulations that can aid the Yemeni people with fundamental human rights. While the most crucial step is to urge the leading Yemeni authorities to restart negotiations to end the war, the more urgent action is to demand the local authorities to join forces in addressing the COVID-19 pandemic by limiting their interferences toward the much-needed humanitarian assistance.

## Financial support

We have not received any financial support for this manuscript.

## Declaration of competing interest

The authors declare no conflicts of interest.
